# Structural Influence on the Post-Clustering Stability of DNA/AgNCs Fluorescence

**DOI:** 10.3390/nano9050667

**Published:** 2019-04-28

**Authors:** Riddhi Nagda, Pratik Shah, Chang Seop Lee, Sooyeon Park, Seong Wook Yang

**Affiliations:** 1 Department of Systems Biology, College of Life Science and Biotechnology, Institute of Life Science and Biotechnology, Yonsei University, Seoul 03722, Korea; riddhinagda@yonsei.ac.kr (R.N.); pshah@yonsei.ac.kr (P.S.); billy961223@gmail.com (C.S.L.); sooypark@yonsei.ac.kr (S.P.); 2 UNIK Center for Synthetic Biology, University of Copenhagen, Thorvaldsensvej 40, 2000 Frederiksberg, Copenhagen, Denmark

**Keywords:** DNA template, silver nanoclusters, nanotechnology, fluorescence, structure, stability

## Abstract

DNA-encapsulated Silver Nanoclusters (DNA/AgNCs) based sensors have gained increasing attention in past years due to their diverse applications in bioimaging, biosensing, and enzymatic assays. Given the potential of DNA/AgNCs for practical applications, the systematic studies of the fluorescent stability over an extended period is necessary. However, the correlation between nucleic acid properties and the long-term stability of DNA/AgNCs is less known. With locking-to-unlocking sensors, in which the secondary structure of DNA template is standardized, we investigated the correlation between the DNA structure and the fluorescence stability of AgNCs. Post-synthesis of DNA/AgNCs, the fluorescence, and structures of templates were monitored over three weeks. By combining the fluorescence spectroscopy with the in-gel fluorescent assay, we found that AgNCs encapsulated by dimer-structured DNA/AgNCs templates were more stable than those of hairpin-structured DNA/AgNCs templates. While the orange fluorescence from the dimer templates increased over three weeks, the red fluorescence from the hairpin templates was diminished by >80% within two days at room temperature. Further tests revealed that hairpin-encapsulated red-emissive AgNCs is more sensitive to oxidation by atmospheric oxygen compared to dimer encapsulated orange AgNCs. Our observations may provide an important clue in encapsulating photophysically more stable AgNCs by tuning the DNA secondary structures. The proposed strategy here can be essential for pragmatic applications of DNA/AgNCs templates.

## 1. Introduction

In recent years, the applications of the fluorescent properties of AgNCs have been increasing at a staggering rate. The applications range from detection of nucleic acids, proteins, and small molecules, to the imaging of these bio-molecules in living cells [1,2,3,4,5,6,7,8,9]. The technologies developed using the photophysical properties of AgNCs for the detection of pathogens, single nucleotide polymorphism and microRNA biomarkers have also been suggested for clinical diagnostics [10,11,12,13,14]. To optimize these DNA/AgNCs based sensing methods, many studies have defined chemical and physical factors of DNA template that modulate the fluorescent properties of DNA/AgNCs. For instance, the sequences of DNA template, secondary structures of DNA, Guanine proximity and flexibility of nucleic acid templates are among the important determinants of emission wavelength and fluorescent intensity [11,15,16,17,18,19]. Specific DNA sequences are also shown to increase the stability of emissive DNA/AgNCs [20,21,22]. Sharma et al. showed that certain DNA sequences could retain almost 31% of the initial fluorescence after almost ten months [20]. However, the properties of the DNA template, beyond sequence, that influence the long-term stability of DNA/AgNCs remain elusive. For a long time, DNA sequence specificity has been emphasized as an important parameter in modulating the properties of AgNCs [18]. However, we previously showed that when a specific DNA sequence encapsulating AgNCs is extended or combined with additional DNA sequences irrelevant to encapsulating AgNCs, the fluorescent properties of the DNA/AgNCs, such as emission wavelength, are dramatically altered [23]. Furthermore, we recently reported that if the secondary structure of a DNA template is given, either a dimer or hairpin, it uniformly determines the emission colors, of the DNA template. When a DNA/AgNCs template forms dimer structure, it prefers to encapsulate orange emissive AgNCs, while when a template forms a hairpin structure, it prefers to encapsulate red emissive AgNCs [24]. Further multiple previous studies have indicated that the red emissive AgNCs are more prone to oxidation by O_2_ [21,25]. Especially, Patrick et al. have shown that fluorescence of red emissive AgNCs is less stable compared to green emissive AgNCs when incubated for longer duration post-synthesis [21]. However, the correlation has not yet been elucidated between various DNA structures and the stability of DNA/AgNCs. With the standardization of DNA structure using locking-to-unlocking DNA sensor design, it is possible to rationally design the DNA structure to encapsulate red and orange emissive AgNCs. Therefore, we investigated whether the secondary structure of a DNA template influences the stability of emissive AgNCs. Indeed, we found that the dimer form of a DNA/AgNCs template is more stable than the hairpin form of the DNA/AgNCs template. Based on this result, we introduce a new notion that the two DNA secondary structures, dimer or hairpin, can be a determinant for the long-term stability of AgNCs post-synthesis.

## 2. Material and Methods

Oligo and Chemical: DNA templates were obtained from mBiotech- an IDT company (Integrated DNA Technologies, BVBA. Interleuvenlaan 12A, 3001 Leuven, Belgium). The synthesis of emissive AgNCs was carried out using AgNO_3_ (>99.99%) and NaBH_4_ (99.99%) from Sigma–Aldrich. A sodium nitrate solution and a Tris-Acetate buffer (pH 7, 0.5 M) from TRIZMA® acetate salt (≥99.0%, from Sigma–Aldrich) were prepared in pure Milli-Q water (18.2 MΩ·cm). The 40% Acrylamide: Bis-acrylamide (19:1) mixture was obtained from Intron Biotechnology (Cat. No. BA003). Ammonium persulphate was obtained from Sigma Aldrich (Yongin City, Korea), while TEMED (tetramethylethylenediamine) was obtained from Bio-Rad (Hong Kong, China). 

Synthesis of AgNCs: To make fluorescent AgNCs, we incubate the DNA probes (25 μL) at 95 °C for 10 min (denaturation) and 25 °C for 20 min (annealing) in the given concentrations of 20 mM Tris-acetate buffer and 25 mM NaNO3. Then, AgNO_3_ and NaBH_4_ were added to the final concentration of 250 μM in 50 μL reaction volume. In addition to the DNA/AgNCs of interest, the synthesis procedure inevitably leads to the formation of silver nanoparticles with absorbance in the 400 nm range as well as minor fluorescent species. During sample preparation, all operations should be performed in a systematic manner to obtain optimal results. Throughout, we designated the concentrations of nucleic acids, buffer, and salts in the final reaction volume (50 μL). All the DNA/AgNCs were incubated for 1 h at 25 °C and subsequently used for further analysis. To measure emission and excitation spectra, the samples were diluted to 200 μL using water. The fluorescence was measured in a 96-well disposable plate using CLARIOstar from BMG Labtech. For long-term study, 45 reactions with each DNA templates were prepared and sealed under ambient conditions. The samples were stored at room temperature in a container. Three samples of each DNA/AgNCs templates were used at respective days for fluorescence measurements and the remaining unused samples were stored again. For fluorescence measurement after re-reduction after 3 months of incubation, 250 μM of freshly prepared NaBH_4_ was added to each sample and the fluorescence was measured within 15 min.

In-gel fluorescence assay: To detect the hairpin structure and self-dimer structures from the DNA/AgNCs, gel electrophoresis analysis was performed with a native polyacrylamide gel (15%). A MiniPROTEAN Tetra Cell system (Bio-Rad) was used for the gel electrophoresis with a TBE buffer (Tris base; 44.5 mM, Boric acid; 44.5 mM, EDTA (Ethylenediaminetetraacetic acid; 1 mM). Gel electrophoresis experiments were performed starting from different samples. The AgNCs samples prepared using the details mentioned before in a 50 μL reaction volume. 25 μL of the reaction mixture was mixed with 4.5 μL of 50% glycerol and 0.5 μL of 1000X SYBR Gold dye (SYBR™ Gold Nucleic Acid Gel Stain, Thermofisher Scientific S11494) for samples detected using Sybr Gold and AgNCs. For AgNCs samples detected without using Sybr Gold, the dye was replaced with distilled water. The electrophoresis was performed at 100 V/1.5 h followed by visualization using Transilluminator. The images were captured using canon camera (EOS 750D, EF-S 18–55 mm lens, under UV light. All the captured images were treated further equally such that it may not result in the altered appearance. For tube images, same tubes were maintained for taking pictures over the extended incubation period at respective days.

## 3. Result and Discussion

**Figure 1:**
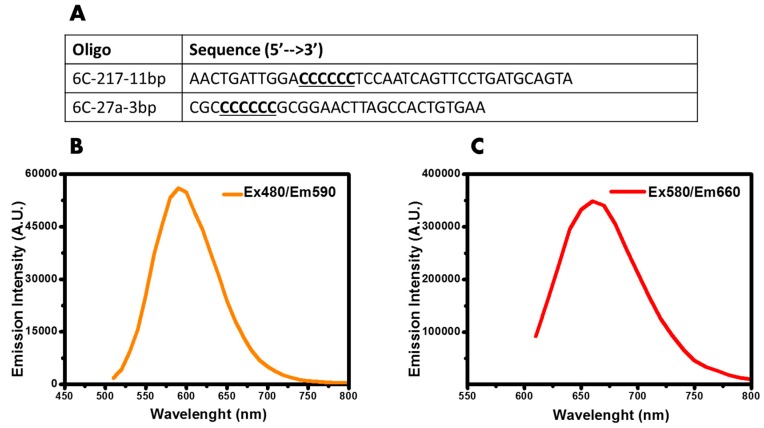
(**A**) Name and 5’ to 3’ sequence of the DNA templates used in the study. (**B**) Fluorescence of DNA 6C-217-11bp with Ex/Em wavelength 480/590 nm. The fluorescence was measured after 60 min of synthesis. (**C**) Fluorescence of DNA 6C-27a-3bp with Ex/Em wavelength 580/660 nm. The fluorescence was measured after 60mins of synthesis.

Previously, we suggested a strategy for DNA/AgNCs template designing, called the locking-to-unlocking sensor system [13]. The advantage of this design includes the flexibility of the sensor to accommodate a variety of target sensing sequences with defined DNA secondary structures. It consists of three components: (i) cytosine-rich loop, (ii) target sensing sequence, and (iii) an anchor sequence which facilitates a fold-back secondary structure. The anchor length needs to be long enough to hold the locking-to-unlocking sensor in the absence of target nucleic acid but weak enough to be disrupted when the complementary sequence is detected. Therefore, depending on the GC content of the sequence, anchor lengths can be modulated which allows this sensor design to be universal for the detection of various miRNA sequences as demonstrated previously [13]. Moreover, we showed that the emission wavelength of the encapsulated AgNCs could be modulated by altering the secondary structure of DNA/AgNCs [24]. Thus, we investigated whether the secondary structure of DNA/AgNCs influences the photo-stability of emissive AgNCs. Using DNA template shown in Figure 1A, we confirmed our previous observation, the structure dependent emission color of DNA/AgNCs–a dimer DNA/AgNCs shows orange, while a hairpin DNA/AgNCs shows red. DNA 6C-217-11bp generated orange emission with Ex/Em wavelength of 480/590 nm (Figure 1B). In contrast, DNA 6C-27a-3bp generated single red emissive AgNCs with Ex/Em of 580/660 nm (Figure 1C). Furthermore, the intensity of DNA 6C-27a-3bp fluorescence was notably higher than that of DNA 6C-217-11bp (Figure 1). 

**Figure 2:**
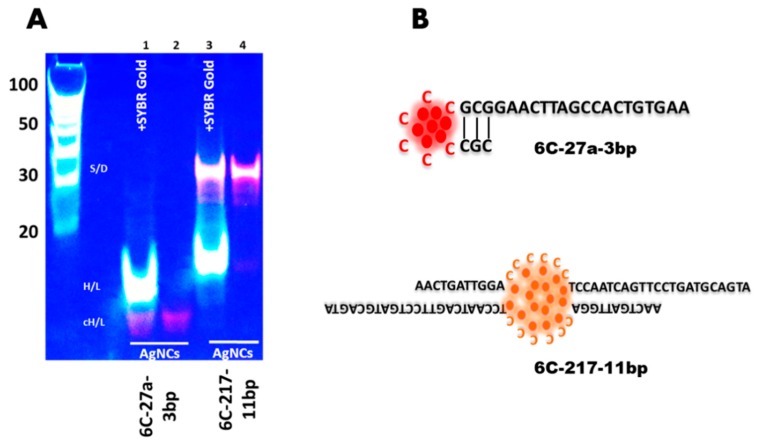
(**A**) Native gel electrophoresis of DNA 6C-27a-3bp/AgNCs and DNA 6C-217-11bp/AgNCs. Samples were prepared with or without SYBR Gold dye. The DNA bands were visualized either with SYBR Gold (SG) and/or native AgNCs fluorescence. The abbreviations cH/L- compact hairpin-loop or SD: self-dimer DNA or H/L: hairpin-loop describes the predicted DNA structures. DNA Ladder on the left shows 100bp size marker. (**B**) Schematic representation of the secondary structures of DNA 6C-27a-3bp and DNA 6C-217-11bp.

Based on our previous study [13,24], we further analyzed the secondary structures of DNA/AgNCs templates using in-gel fluorescence assay, to see whether the secondary structures are correlated to the color of fluorescence. Indeed, we found that the secondary structure of DNA 6C-217-11bp is a dimer, while DNA 6C-27a-3bp generated a hairpin structure (Figure 2). We used SYBR Gold to visualize the presence of all the DNA structures which may or may not encapsulate AgNCs. A specific red or orange fluorescence in the absence of SYBR Gold helps to identify specific DNA structures, encapsulating emissive AgNCs. In lane 1, we added SYBR Gold to show all the structures of DNA 6C-27a-3bp template with AgNCs. Moreover, we observed that when the DNA 6C-27a-3bp template encapsulated red emissive AgNCs, the hairpin structure became more compact and migrated much faster than the normal hairpin structure of DNA 6C-27a-3bp without AgNCs (Figure 2, lane 1). Without SYBR Gold, the red fluorescence from a hairpin-structured DNA 6C-27a-3bp template was clearly seen in lane 2. In lane 3 and 4, we observed the profoundly shifted orange bands that caused by dimerization of DNA 6C-217-11bp template, encapsulating orange emissive AgNCs. DNA 6C-217-11bp hairpin structure without encapsulated AgNCs was stained with SYBR Gold as can be seen in lane 3 but not in lane 4. The orange fluorescence from a dimer structured DNA 6C-217-11bp template was seen without SYBR Gold (Figure 2, lane 4). Previous studies reported a similar transformation of red and non-red emission in i-motif structure [26,27]. It is especially interesting to note the results of the Sugimoto group here. Even though their designing strategy was different to ours, their sensor was hybridized with target sequence forming dimer structure, and the AgNCs encapsulated in i-motif structure generated orange emission. In contrast, if the DNA sequence was not hybridized with a target sequence resulted in red emission [26]. Next, we examined the photo-stability of AgNCs in these templates to see if there is any correlation between the secondary structure of the DNA template and the stability of synthesized AgNCs.

**Figure 3:**
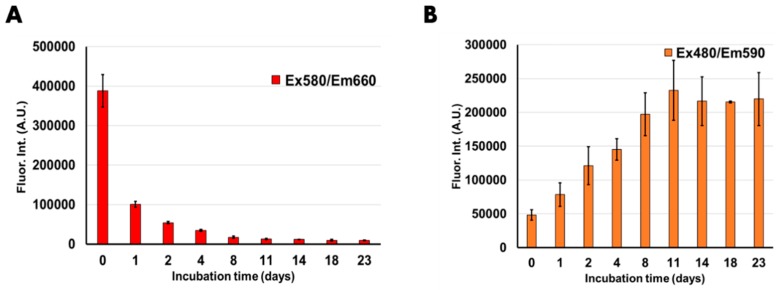
The relative emission intensity of (**A**) DNA 6C-27a-3bp/AgNCs and (**B**) DNA 6C-217-11bp/AgNCs over 23 days with Ex/Em of 580/660 nm and 480/590 nm respectively. Day 0 refers to the measurement of fluorescence emission after 1 h of synthesis. All the DNA/AgNCs samples were prepared on Day 0 and at designated day each Eppendorf tube was used for measurements. The samples were stored at room temperature and were prepared under ambient conditions. The error bars refer to standard deviation obtained from three independent replicates.

To comparatively measure the fluorescence intensity in a time-dependent manner, we synthesized 45 samples of each DNA/AgNCs template on day 0. The fluorescence consistency for all the prepared samples was visualized in Supplementary Figure S1. On each designated day, three samples of each DNA/AgNCs template were used for fluorescence measurement, and the average emission intensity has been shown in Figure 3. The error bars demonstrate the standard deviation among the measured samples. First, we measured the fluorescence of DNA/AgNCs template at one hour on the day of synthesis (day 0) after encapsulating AgNCs as a control point. Then, to ensure the fluorescence specificity, we determined the fluorescence emission spectra of both DNA templates across UV-Visible wavelengths (Supplementary Figure S2). After 24 h (1 day) incubation, the red fluorescence of DNA 6C-27a-3bp was reduced by over 75%. After two-day incubation, the red fluorescence was further reduced by over 85%. By day 4, the red emission was reduced by over 90% as compared to day 0, and the remaining 10% fluorescence continued to diminish further until day 23. At the end of incubation, only about 2% red fluorescence was retained. Long-term incubation over 23 days, the red fluorescence of DNA 6C-27a-3bp was reduced by almost 98% compared with the 0-day control (Figure 3A). On the other hand, we analyzed the orange emissive AgNCs fluorescence of DNA 6C-217-11bp over 23 days (Figure 3B). The orange emission at day 2 was almost 2.5 fold higher compared to the 0-day control. The fluorescent intensity of DNA 6C-217-11bp increased by 3-fold after a 4-day incubation. The orange fluorescence increased further until day 18 and remained steady then until day 23. At the end of 23- day measurement, the orange fluorescence increased by over 400% of the fluorescence intensity of the 0-day control, whereas the red emissive AgNCs of DNA 6C-27a-3bp lost 98% of the original fluorescence intensity.

**Figure 4:**
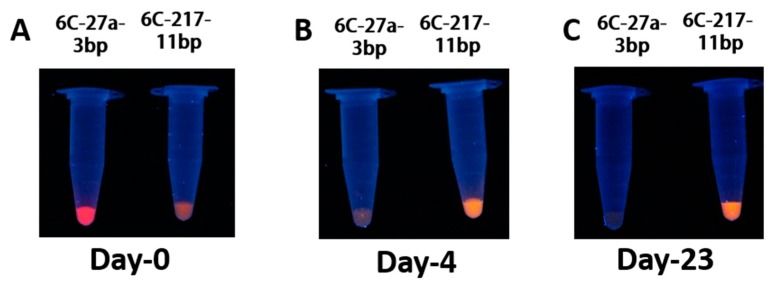
(**A**–**C**) Eppendorf tube images under UV (Ex 365 nm) illumination. All the samples were prepared on day 0 and were stored before taking images at designated time (in days) post synthesis. The pictures were taken following similar camera set up as mentioned previously.

Previously, it was shown that the red-emissive AgNCs is highly prone to oxidation by O_2_ in the air when incubated post-synthesis for an extended period [21,25]. Patrick et al. have shown that fluorescence of red emissive AgNCs decreases quickly compared to green emissive AgNCs when incubated for longer duration post-synthesis [21]. Further, the blue-shifting of red-emissive AgNCs was reported due to oxidation by atmospheric oxygen [25]. Therefore, to see any possible emission shift of emissive AgNCs during the incubation period, we monitored the full scan of emission spectrum DNA/AgNCs (Supplementary Figures S3–S9). Interestingly, we observed the formation of yellow emissive AgNCs in the DNA 6C-27a-3bp, while its red emissive AgNCs was gradually diminished as the incubation time passes. This blue shift of red-emissive AgNCs is consistent with the previous report [25]. Next, we confirmed the fluorescent patterns of DNA 6C-27a-3bp and DNA 6C-217-11bp templates using UV illumination. As shown in Figure 4A, the red color of DNA 6C-27a-3bp and the orange color of DNA 6C-217-11bp can be seen right after one hour of AgNCs clustering. Consistent with the fluorescence spectroscopic analysis (Figure 3), the red emissive AgNCs of DNA 6C-27a-3bp rapidly decreased after two days, whereas the orange-emitting AgNCs of DNA 6C-217-11bp increased and then remained unchanged even after 23 days (Figure 4B,C). Based on our result and previous literature, we suggest that the non-red emission, in the case of orange emission, seems to be highly stable and resistant to further oxidation. Moreover, the red emission seems to be relatively unstable and prone to oxidation over time. These results indicated that the orange emission from a dimer structure was highly stable than the red emission from a hairpin structure, regardless of their initial emission intensity (Table 1). 

**Figure 5:**
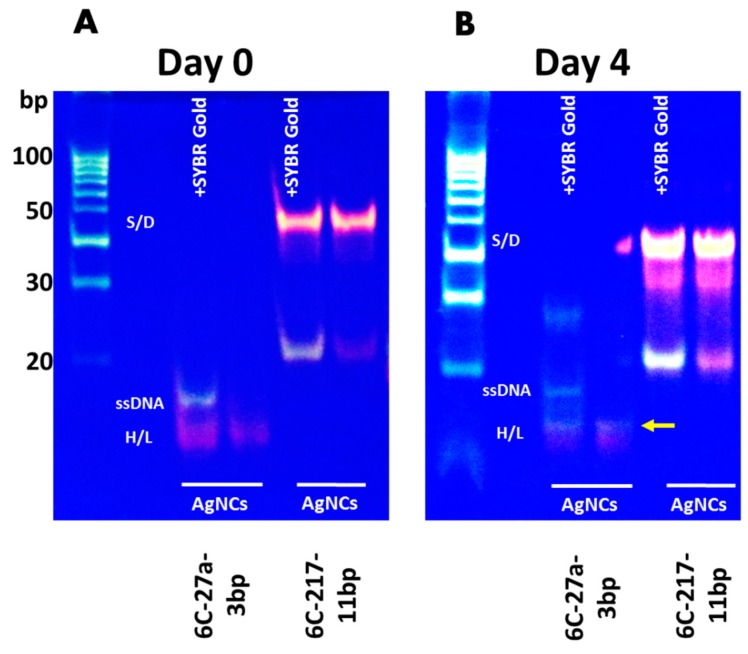
Native gel electrophoresis of (**A**) DNA 6C-27a-3bp/AgNCs and (**B**) DNA 6C-217-11bp/AgNCs performed after 1 h on day 0 and after 4 days post-AgNCs synthesis. Before running the gel, samples were prepared with or without SYBR Gold dye. The DNA bands were visualized either with SYBR Gold (SG) and/or native AgNCs fluorescence. Yellow emission of DNA 6C-27a-3bp/AgNCs on day-4 is indicated with arrow. The abbreviations cH/L- compact hairpin-loop or SD: self-dimer DNA or H/L: hairpin-loop describes the predicted DNA structures. DNA Ladder on the left shows 100bp size marker.

**Figure 6:**
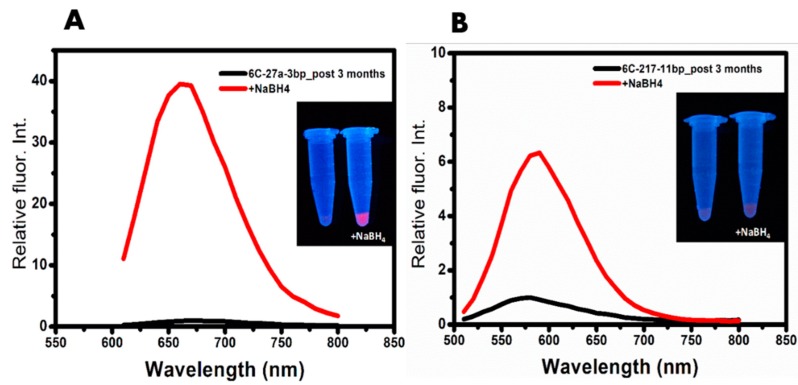
(**A**) Emission wavelength of DNA 6C-27a-3bp with Ex/Em wavelength 580/660 nm. (**B**) Emission wavelength of DNA 6C-217-11bp with Ex/Em wavelength 480/590 nm. Insets show the eppendorf tube images under UV (Ex 365 nm) illumination before and post NaBH_4_ additions. Black line refers to fluorescence of DNA/AgNCs post 3 months of incubation, while red line refers to re-reduced DNA/AgNCs fluorescence with 250 μM NaBH_4_.

Next, we investigated whether the DNA templates show any changes in their secondary structures during the incubation period (Figure 5). All the AgNCs samples were synthesized on day 0. The in-gel fluorescence assay was performed on day 0 and day 4. Day 4 was selected since DNA 6C-217-11bp showed three-fold higher emission compared to Day 0 while DNA 6C-27a-3bp lost >90% of initial emission intensity. The samples were resolved by native gel electrophoresis with or without SYBR Gold stain. By the addition of SYBR Gold, the presence of all DNA bands was shown irrespective of the encapsulating of AgNCs. Besides, the detection of specific fluorescence by AgNCs confirmed the presence of DNA structure encapsulating AgNCs. With this analysis, we aimed to determine if the alternation of fluorescence is caused by the structural changes of DNA templates or the altered photophysical properties of AgNCs during an extended incubation period. We observed that on 0-day, DNA 6C-27a-3bp encapsulated red emissive species that form a compact hairpin-loop structure (cH/L) which migrates faster than the hairpin form of DNA 6C-27a-3bp template without AgNCs, possibly due to a structural tangling gained through the encapsulation of AgNCs. We found that SYBR Gold was unable to intercalate the compact hairpin structure by encapsulated AgNCs, possibly due to the structural tangling by AgNCs. Contrastly, SYBR Gold intercalated the non-clustered hairpin DNA template. This phenomenon also implies that the place of AgNCs clustering in the hairpin DNA template could overlap with the intercalation place of SYBR Gold. However, a detailed study of this phenomenon should be further commenced. On 4-day, both the cH/L and hairpin DNA templates were stained with SYBR Gold and the fluorescence originated from AgNCs was almost diminished. We observed the appearance of yellow emissive AgNCs in the upper position of the cH/L of DNA 6C-27a-3bp in the gel. This result was consistent with the fluorescence emission data (supplementary Figure S5) and implied that a structural shift might be related to the transformation of red emissive AgNCs to yellow emission during prolonged incubation post-synthesis (Figure 5B). DNA 6C-217-11bp forms a distinctive dimer structure that migrated slower than the hairpin DNA template without AgNCs on 0-day (Figure 5A, lane 3). In addition to the orange emissive AgNCs, we observed the presence of a small amount of red emissive hairpin DNA 6C-217-11bp. After four days of incubation post-AgNCs synthesis, the orange emission of 6C-217-11bp increased, while the lament amount of red emissive AgNCs became yellowish (Figure 5B, lane 4). To investigate the underlying mechanism that led to the loss of red fluorescence while retaining strong orange fluorescence, we further incubated samples for three additional months at room temperature. During the incubation period, orange emission also dissipated completely. We hypothesized that the red emissive AgNCs could be more prone to oxidation orange AgNCs. If the hypothesis related to oxidation of AgNCs is correct, the re-reduction process could restore red fluorescence. As shown in Figure 6, we observed that the red emission of AgNCs increased by over 40-fold by adding additional NaBH4 to 6C-27a-3bp after three months of incubation. Moreover, the dissipation of orange fluorescence took over three months, but the restoration of orange fluorescence was less effective in DNA 6C-217-11bp dimer (Figure 6B). These results indicated that the loss of red fluorescence by over 90% within four days of incubation was mostly due to oxidation. The re-gaining of red fluorescence by re-reduction was consistent with previous reports [22,25]. Taken together, we suggest that the dimer DNA structures could protect encapsulated AgNCs from rapid oxidation more efficiently than hairpin DNA structures. 

## 4. Conclusions and Future Perspective

Many parameters affecting the generation and stability of DNA-encapsulated AgNCs (DNA/AgNCs) have been identified such as pH, oxidation, and buffer conditions, along with DNA sequences [15,17,18,25]. However, except for several systematic attempts [20,21,22,25], the fluorescent properties of AgNCs post-synthesis are less known over prolonged incubation. The effect of DNA secondary structure on the stability of AgNCs fluorescence is not fully understood. In this direction, we showed that there is a possible correlation between the template DNA structure and the stability of encapsulated AgNCs. Using 6C-217-11bp template encapsulating orange emissive AgNCs, we observed that fluorescence of DNA/AgNCs was stable for up to 23 days with close to five-fold higher emission compared to the day of synthesis. The AgNCs appeared to be unaffected by any oxidation. Moreover, red emissive AgNCs encapsulated by DNA template 6C-27a-3bp lost its fluorescence over 90% within four days of synthesis. The red emission was reduced by about 98% by day 23. Taken together, our data indicate that dimer structure is more important in the generation of highly stable orange emissive AgNCs while the hairpin encapsulating red-emissive AgNCs is less stable. It took three months for dimer structure to lose nearly all fluorescence which took only four days for the hairpin structure. Further re-reduction and regain of the AgNCs fluorescence in the case of DNA 6C-27a-3bp after three months indicated the loss of fluorescence was due to oxidation of AgNCs. Therefore, we suggest here that there is an inherent difference in DNA structures for protecting the encapsulated AgNCs post-synthesis from oxidation. It is proposed here that hairpin encapsulated AgNCs could be more sensitive to oxidation than dimer encapsulated AgNCs which may lead to the higher stability of dimer encapsulated orange emissive AgNCs compared to red emissive AgNCs encapsulated in a hairpin structure. 

This study indicates that it might be useful to design dimer generating DNA/AgNCs template for the applications where long-term stability of fluorescent AgNCs post-synthesis is sought. However, more of these photo-stability studies are needed to understand how DNA secondary structures influence the photo-stability of AgNCs. For instance, two important questions on the correlations between DNA secondary structures and the photo-stability of AgNCs should be answered: Why does DNA dimer structure encapsulate more stable AgNCs? Can the structural shift to a hairpin weaken the AgNCs stability in the dimer? Seeking the answers to the questions through detailed structural analyses will be the next challenge.

## Figures and Tables

**Table 1:** Summarized result of emission intensities of DNA 6C-27a-3bp/AgNCs and DNA 6C-217-11bp/AgNCs after designated incubation time post-synthesis of DNA/AgNCs. % Emission Intensity refers to the amount of fluorescence remained after given number of days compared to 1 h (day 0) post- AgNCs synthesis. Emission intensities mentioned are the average of three independent samples prepared concurrently. S.D. refers to standard deviation obtained from three independent replicates.

Incubation Period (Post-AgNC Synthesis)	DNA 6C-27a-3bp/AgNCs (Ex/Em—580/660 nm)		DNA 6C-217-11bp/AgNCs (Ex/Em—480/590 nm)	
	Emission Intensity (A.U.) ± S.D.	% Emission Intensity	Emission Intensity (A.U.) ± S.D.	% Emission Intensity
Day 0 (1 h)	388,094 ± 41,093	100	48,202 ± 7755	100
Day 1 (24 h)	100,834 ± 7389	25.98	78,551 ± 17,451	162.96
Day 2	54,288 ± 2711	13.99	121,255 ± 28,110	251.56
Day 4	34,599 ± 2601	8.92	145,206 ± 15,778	301.24
Day 8	17,338 ± 2794	4.47	197,411 ± 31,692	409.55
Day 11	12,832 ± 1642	3.31	232,639 ± 44,227	482.63
Day 23	9422 ± 493	2.43	219,815 ± 39,173	456.02

## Supplementary Materials

The following are available online at https://www.mdpi.com/2079-4991/9/5/667/s1, Figure S1: Eppendorf tube images under UV (Ex 365 nm) illumination. All the samples were prepared on day 0 and were taken just after and hour of synthesis before storing for long term studies. (A) DNA 6C-27a-3bp/AgNCs and (B) DNA 6C-217-11bp/AgNCs. Figure S2–S9: full scan emission profile of DNA 6C-27a-3bp/AgNCs and DNA 6C-217-11bp/ AgNCs on 0, 1, 2, 4, 8, 11, 18- and 23-days post-synthesis of AgNCs.
