# Supplementary material for: Structural Influence on the Post-Clustering Stability of DNA/AgNCs Fluorescence
Source: Nanomaterials (Basel). 2019 Apr 28;9(5):667. doi: 10.3390/nano9050667 (PMC6566520; doi:10.3390/nano9050667)
Supplement: Supplementary file 1 [file nanomaterials-09-00667-s001.pdf]

Supplementary Information

Structural influence on the post-clustering stability of DNA/AgNCs fluorescence

Riddhi Nagda[1]+, Pratik Shah[1]+, Chang Seop Lee[1], Sooyeon Park[1] and Seong Wook Yang [1,2,*]

1. Department of Systems Biology, College of Life Science and Biotechnology, Institute of Life Science and Biotechnology, Yonsei University, Korea
2. UNIK Center for Synthetic Biology, University of Copenhagen, Thorvaldsensvej 40, DK-1871, Frederiksberg C, Copenhagen, Denmark

\+ Equal Contribution

* Correspondence: yangsw@yonsei.ac.kr;

A B

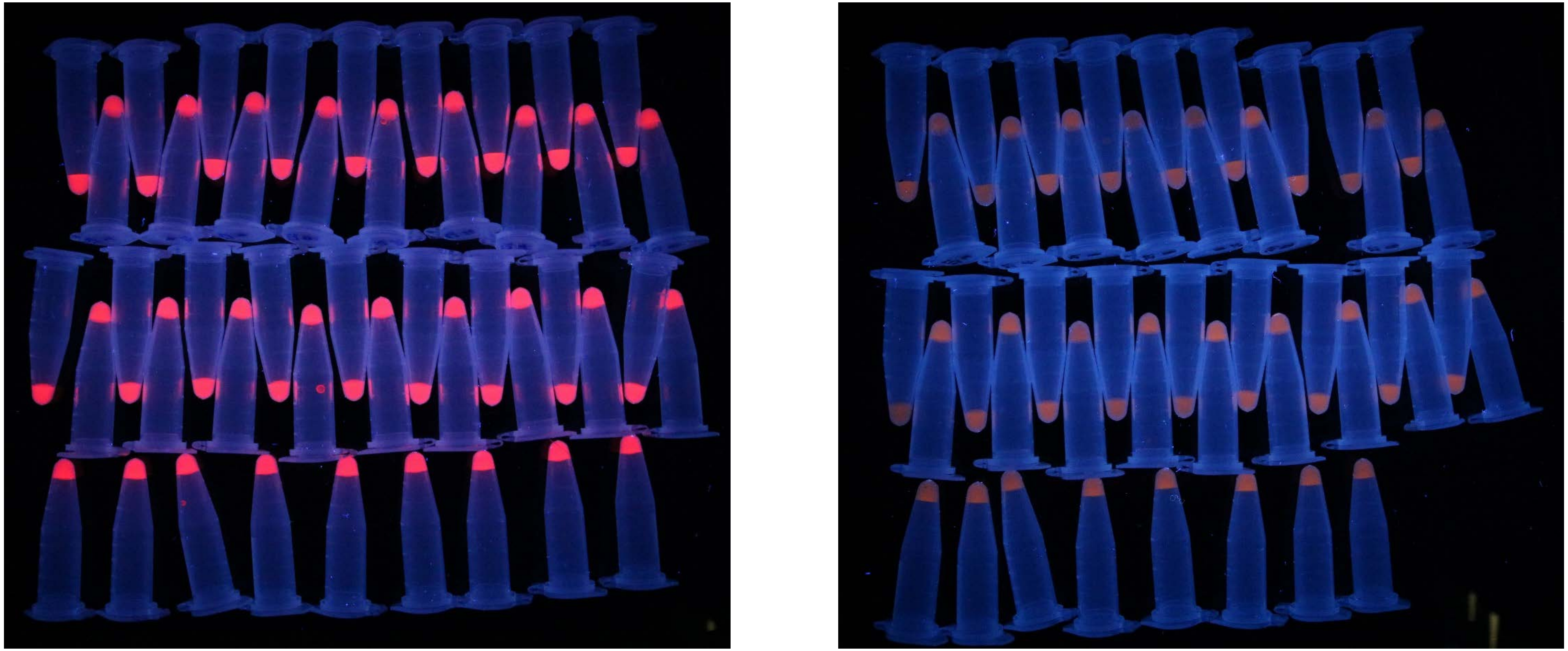

Supplementary Figure 1. Eppendorf tube images under UV (Ex 365nm) illumination. All the samples were prepared on day 0 and were taken just after and hour of synthesis before storing for long term studies. A) DNA 6C-27a-3bp/AgNCs and B) DNA 6C-217-11bp/AgNCs

Day-0

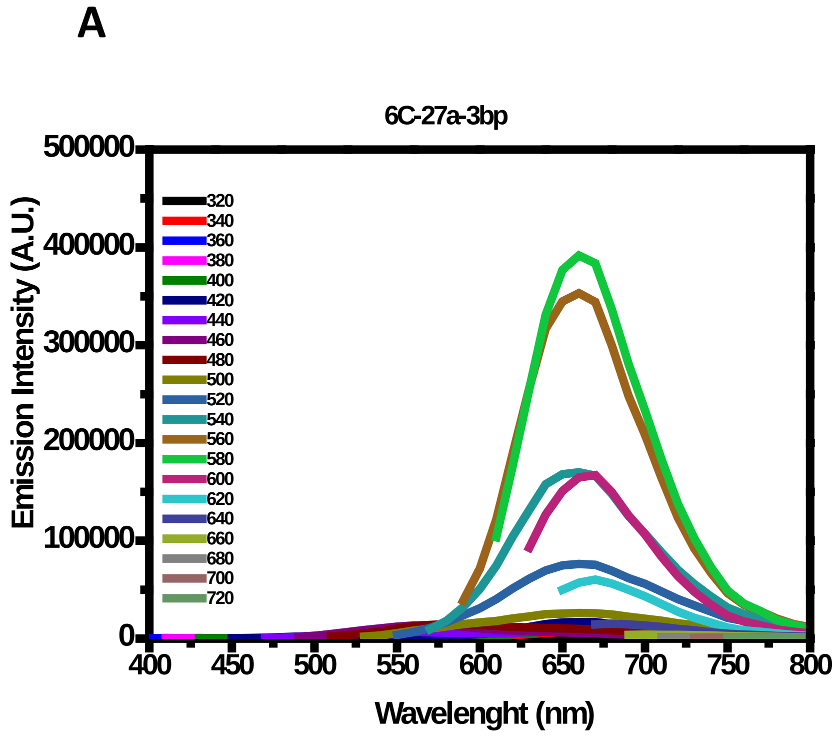

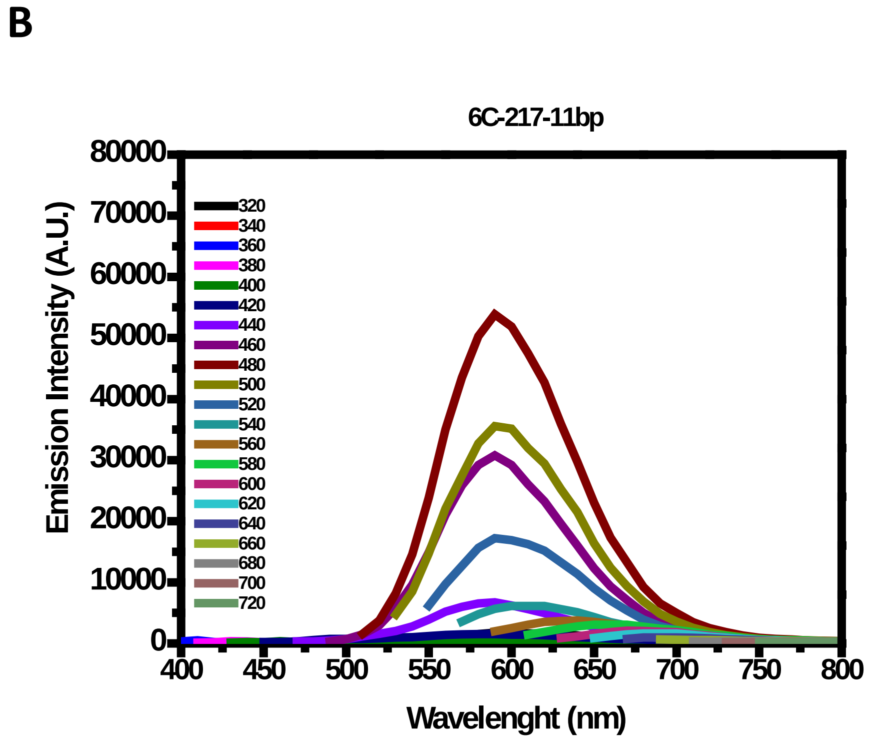

Supplementary Figure 2. Emission spectra of (A) 6C-27a-3bp/ AgNCs and (B) DNA 6C-217-11bp/AgNCs. The spectra were recorded by exciting from 300-720 nm in 20 nm steps after 60mins of post-synthesis referred to as Day 0.

Day-1

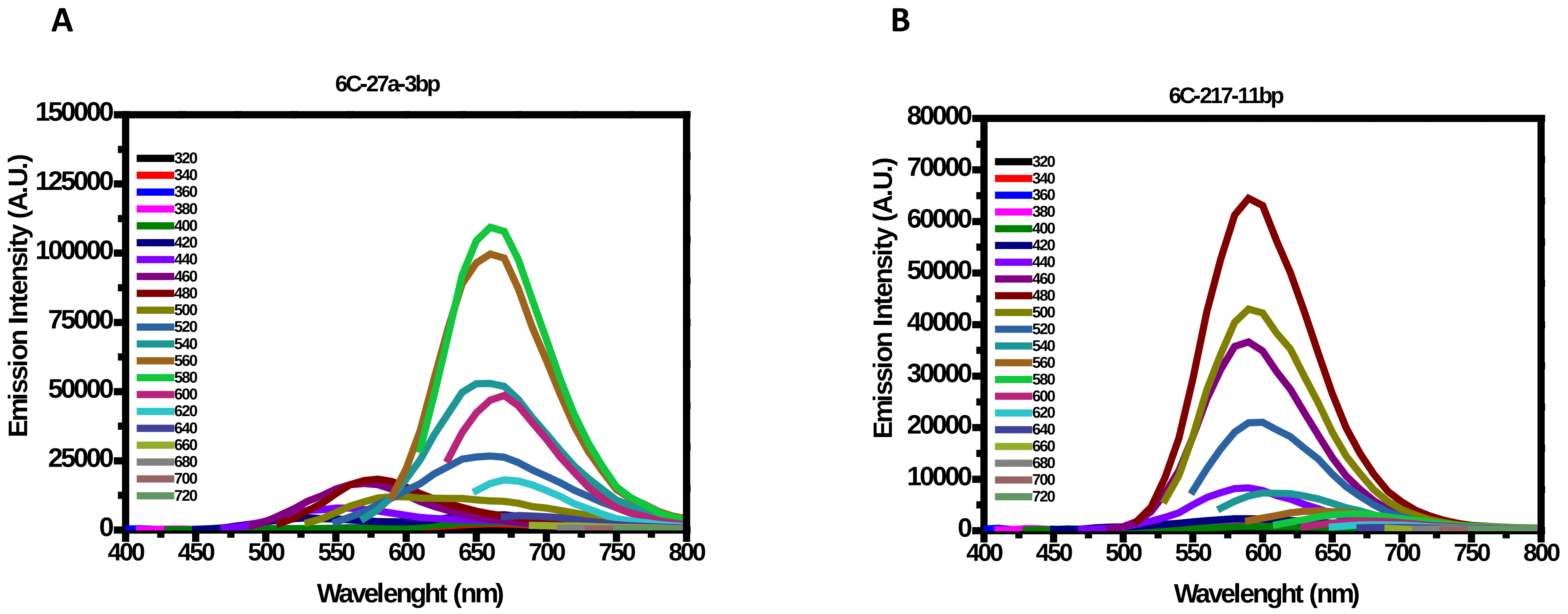

Supplementary Figure 3. Emission spectra of (A) 6C-27a-3bp/ AgNCs and (B) DNA 6C-217-11bp/AgNCs. The spectra were recorded by exciting from 300-720 nm in 20 nm steps after 24hr post-synthesis referred to as Day 1.

Day-2

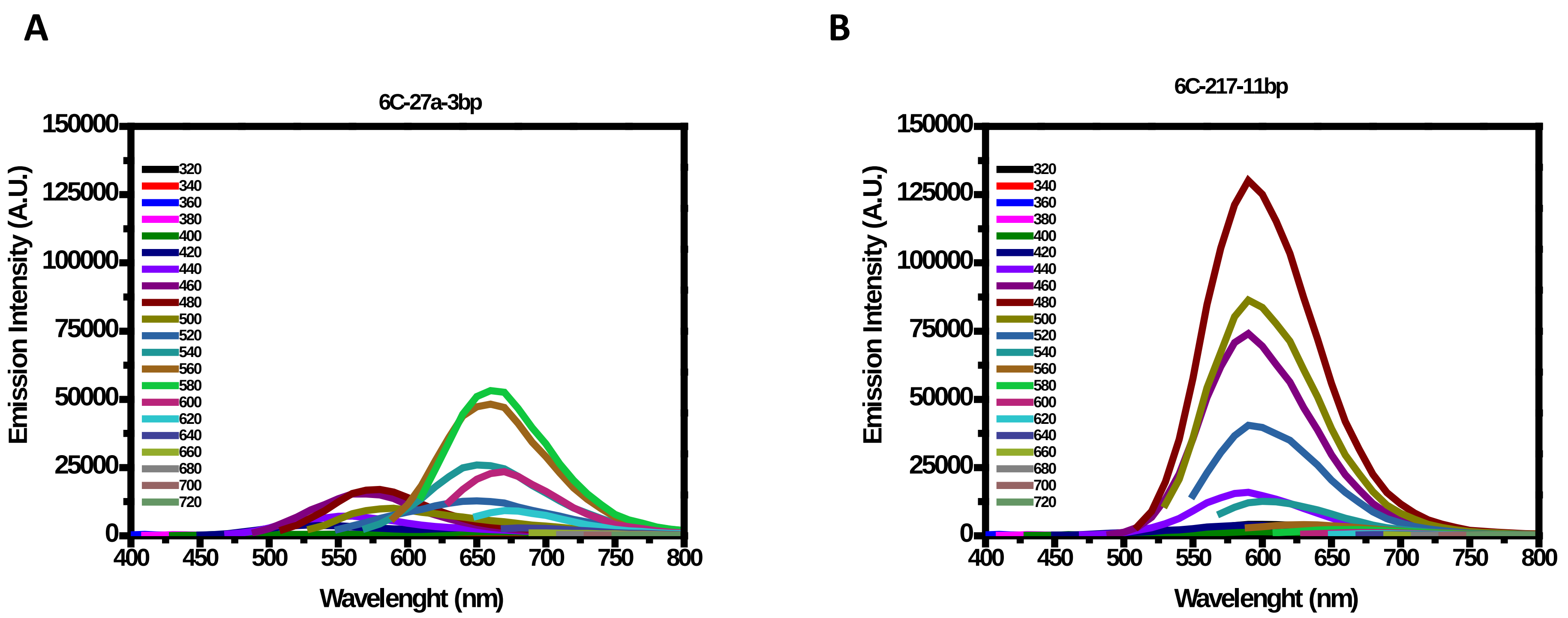

Supplementary Figure 4. Emission spectra of (A) 6C-27a-3bp/ AgNCs and (B) DNA 6C-217-11bp/AgNCs. The spectra were recorded by exciting from 300-720 nm in 20 nm steps after 2 days post-synthesis.

Day-4

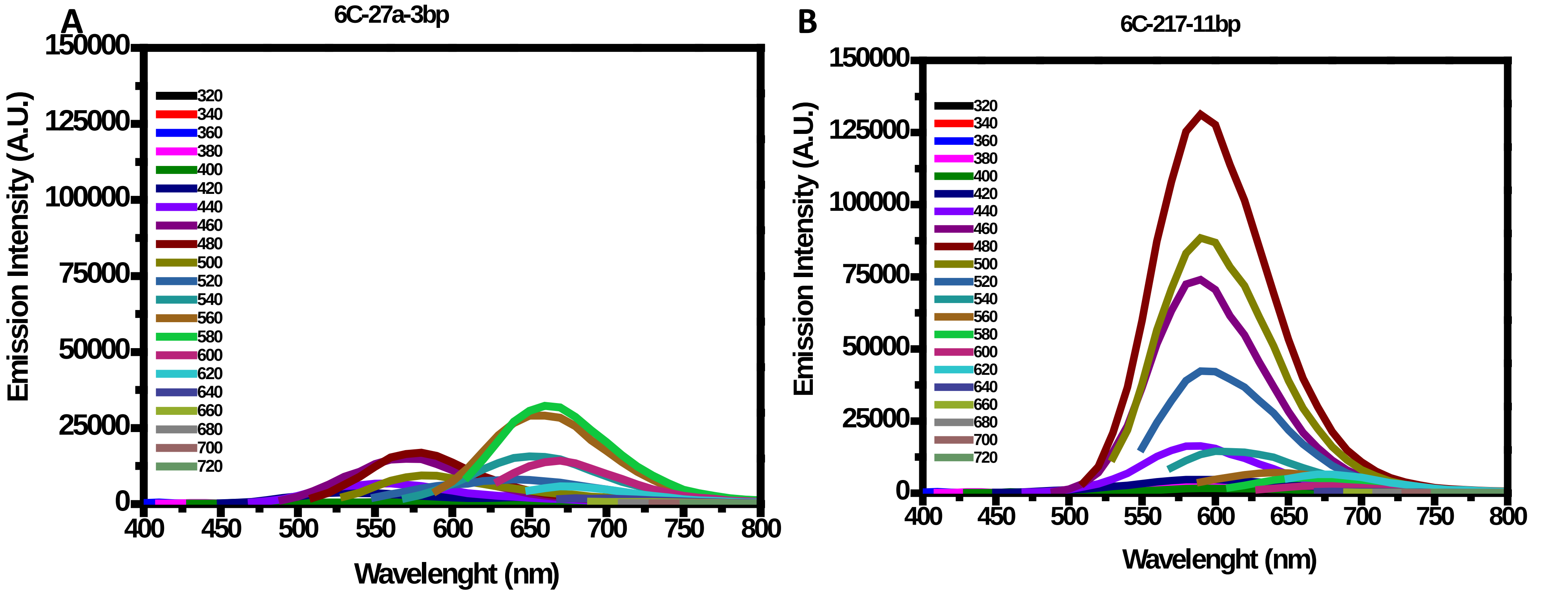

Supplementary Figure 5. Emission spectra of (A) 6C-27a-3bp/ AgNCs and (B) DNA 6C-217-11bp/AgNCs. The spectra were recorded by exciting from 300-720 nm in 20 nm steps after 4 days post-synthesis.

Day-8

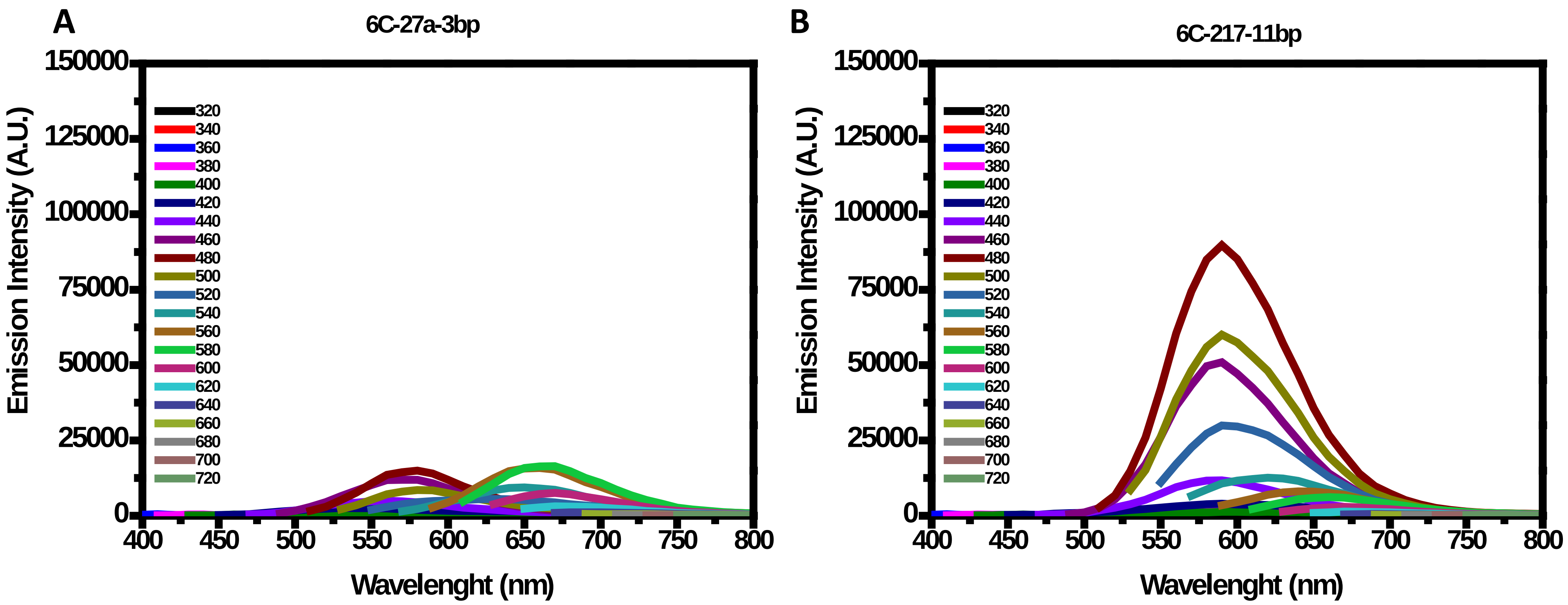

Supplementary Figure 6. Emission spectra of (A) 6C-27a-3bp/ AgNCs and (B) DNA 6C-217-11bp/AgNCs. The spectra were recorded by exciting from 300-720 nm in 20 nm steps after 8 days post-synthesis.

Day-11

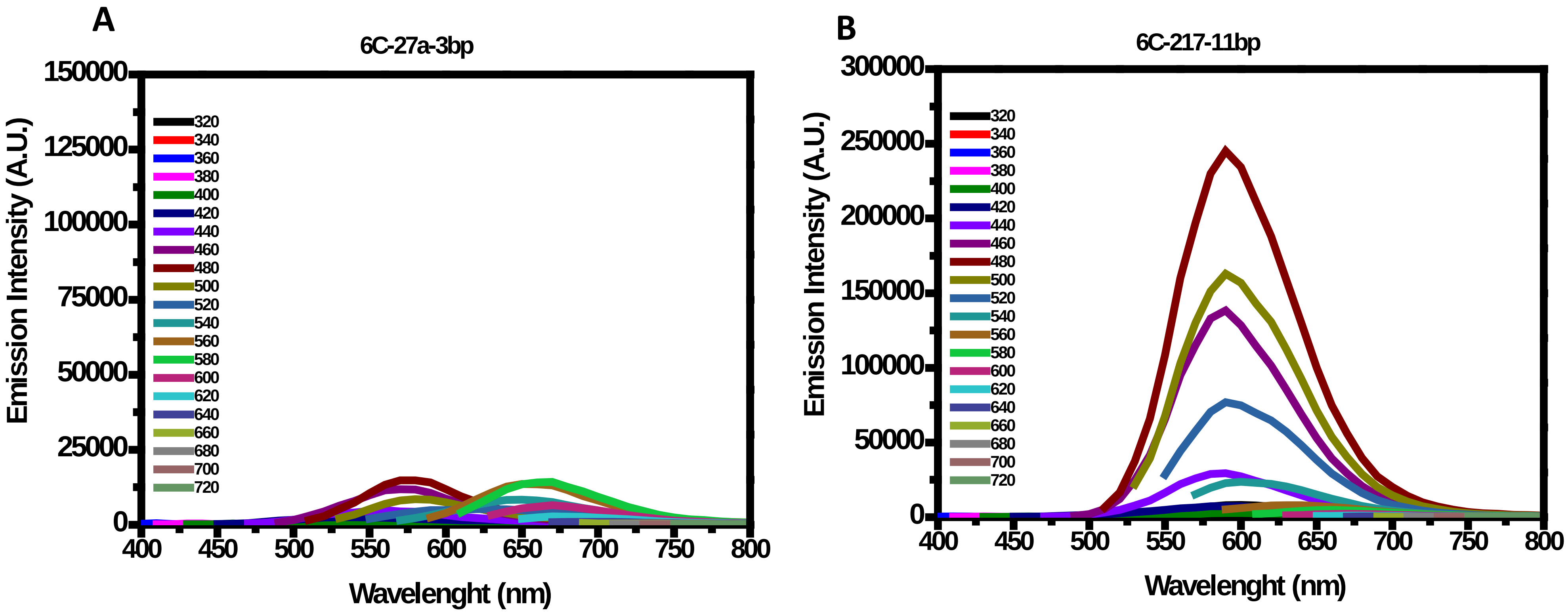

Supplementary Figure 7. Emission spectra of (A) 6C-27a-3bp/ AgNCs and (B) DNA 6C-217-11bp/AgNCs. The spectra were recorded by exciting from 300-720 nm in 20 nm steps after 11 days post-synthesis.

Day-18

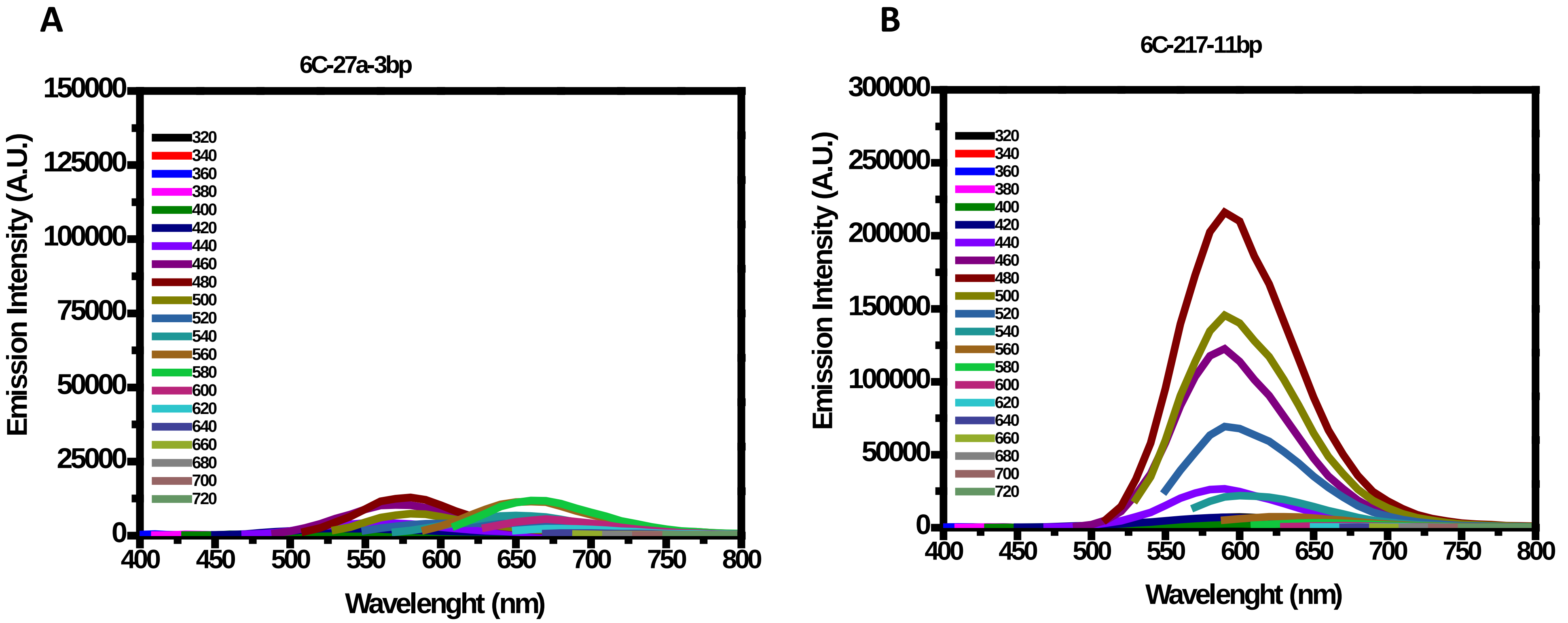

Supplementary Figure 8. Emission spectra of (A) 6C-27a-3bp/ AgNCs and (B) DNA 6C-217-11bp/AgNCs. The spectra were recorded by exciting from 300-720 nm in 20 nm steps after 18 days post-synthesis.

Day-23

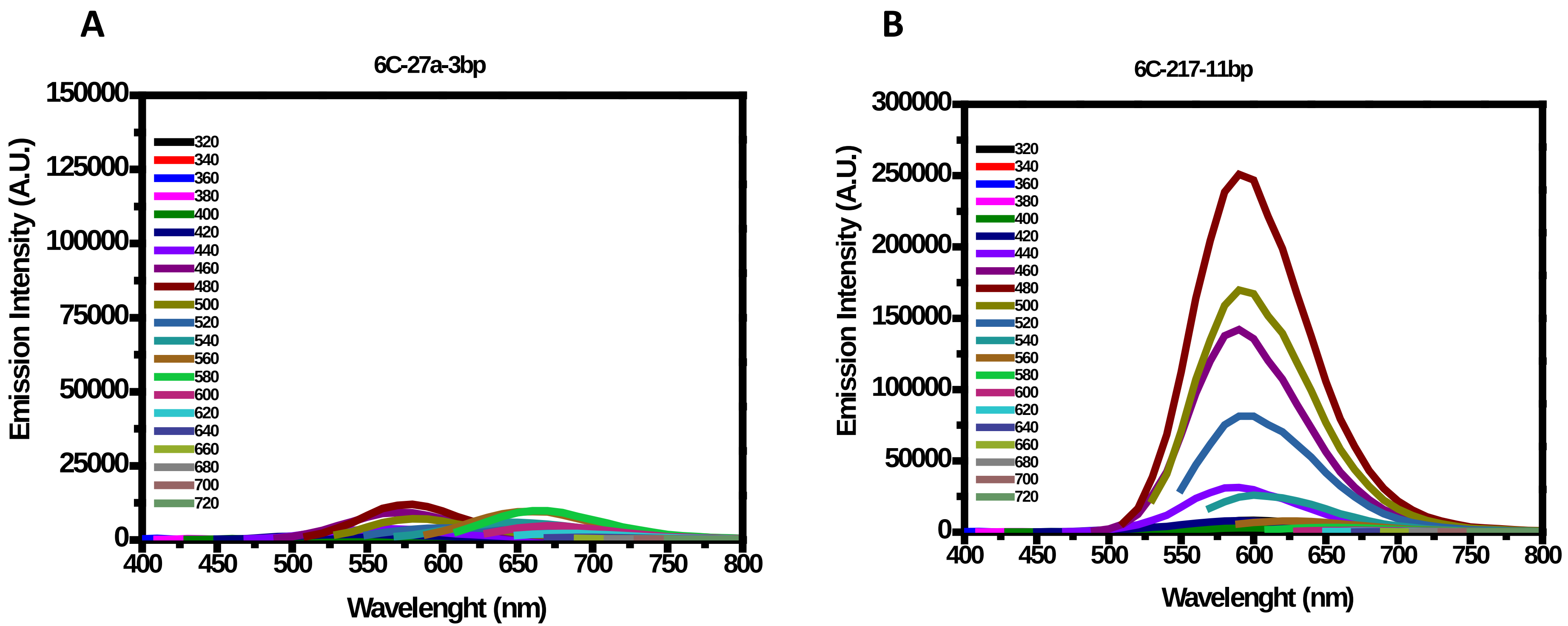

Supplementary Figure 9. Emission spectra of (A) 6C-27a-3bp/ AgNCs and (B) DNA 6C-217-11bp/AgNCs. The spectra were recorded by exciting from 300-720 nm in 20 nm steps after 23 days post-synthesis.
